# Enhanced Lateral Flow Immunoassay for Pesticide Paraquat Based on Combining Magnetite and Gold Nanoparticles

**DOI:** 10.3390/toxics14010002

**Published:** 2025-12-19

**Authors:** Lyubov V. Barshevskaya, Nadezhda A. Taranova, Dmitriy V. Sotnikov, Chuanlai Xu, Anatoly V. Zherdev, Boris B. Dzantiev

**Affiliations:** 1 A.N. Bach Institute of Biochemistry, Research Center of Biotechnology of the Russian Academy of Sciences, Leninsky Prospect 33, 119071 Moscow, Russiasotnikov-d-i@mail.ru (D.V.S.); zherdev@inbi.ras.ru (A.V.Z.); 2 State Key Laboratory of Food Science and Resources, Jiangnan University, Wuxi 214122, China; 3 International Joint Research Laboratory for Biointerface and Biodetection, and School of Food Science and Technology, Jiangnan University, Wuxi 214122, China

**Keywords:** lateral flow assay, magnetite nanoparticles, gold nanoparticles, magnetic concentration, paraquat, food safety control

## Abstract

A lateral flow immunoassay (LFIA) utilizing two types of marker conjugates—magnetite nanoparticles (MNPs) with specific antibodies and gold nanoparticles (GNPs) with anti-species antibodies—was proposed and realized for the detection of the pesticide paraquat. In this assay, the MP conjugate is used to concentrate the target analyte from the tested sample and then to form labeled immune complexes at the test strip, while the GNP conjugate is then applied for the integration into the formed complexes in the binding zone. The magnetic preconcentration allows for working with large volumes of samples, and the following treatment by the GNP conjugate enhances the coloration by five times for reliable analyte revelation in lower concentrations. In the course of the assay implementation, its conditions have been optimized, and the efficiency of the paraquat determination in orange samples was confirmed. The achieved detection limits were 1.2–3.7 ng/mL for visual assessment and 0.12–0.48 ng/mL for the instrumental one, with paraquat detection rates ranging from 96% to 120%. The implementation of the assay in combination with the stage of magnetic concentration allows for the collection of paraquat from large volumes of samples and detects it in this way in concentrations up to two orders of magnitude smaller.

## 1. Introduction

Lateral flow immunoassays (LFIAs) are currently one of the most widespread, rapid, easy-to-use, and convenient tools for rapid analysis. LFIAs have found widespread applications in food safety, allowing for the detection of a wide range of hazardous contaminants in different food matrices (such as milk, meat products, honey, baby food, vegetables, fruits, and grains, etc.) [1]. Target analytes that are commonly monitored in foods include pathogenic microorganisms [2,3,4], mycotoxins [5,6], food allergens [7,8], and antibiotics [9].

An important field in LFIA is the detection of pesticides to ensure the safety of food materials before consumption or further processing. Among the variety of pesticides, paraquat, which belongs to bipyridyl compounds and is used primarily to kill weeds and desiccate crops before harvest, is of particular concern [10,11]. While many countries restrict or ban paraquat, other regions continue to use it [12,13,14,15,16]. The primary danger of paraquat use is its high toxicity. Acute oral paraquat poisoning causes severe damage to the gastrointestinal tract, liver, kidneys, and lungs. The most dangerous is progressive pulmonary fibrosis, leading to respiratory failure and high mortality. Chronic exposure to low doses, including consuming foods with paraquat residues, can lead to nephrotoxicity, hepatotoxicity, and neurological disorders [17,18]. Furthermore, paraquat is poorly degraded in soil (with a half-life of up to 7 years) and can accumulate in agricultural crops and lead to water pollution [19,20].

Therefore, the detection of paraquat residues in food products is essential. The main methods applied for this purpose are high-performance liquid chromatography [21,22], gas chromatography [23,24], and capillary electrophoresis [25,26]. However, the use of these methods is limited by the need for expensive equipment and labor-intensive and time-consuming sample preparation, which complicates the mass screening of large numbers of samples.

Among the immunochemistry methods for paraquat detection, the most used is the enzyme-linked immunosorbent assay (ELISA) [27,28]. There are also developments of immunosensors for the determination of paraquat [29,30,31]. Despite having high sensitivities, these methods are still labor-intensive and time-consuming. On the contrary, LFIA provides high sensitivity, simplicity, and rapidity of detection, so it has the potential to be a prospective and cost-effective tool for mass screening.

Most of the LFIAs utilize only one type of marker nanoparticle (for example, gold nanoparticles) to detect paraquat in different food or biological samples. To improve the analytical characteristics of LFIA, magnetite nanoparticles can be a useful tool. Magnetic nanoparticles modified with functional groups serve as carriers for biomolecules and are capable of selectively binding the antigens in complex matrices [32]. The use of an external magnetic field allows for the concentration of the formed immune complexes, thereby increasing the concentration of the target analyte [33,34]. Furthermore, magnetic preconcentration facilitates the effective removal of matrix impurities and the reduction in background signals, which is important for analyzing biological or food samples with a high content of inhibitory substances [35,36]. Thus, the advantage of using magnetite nanoparticles in LFIA is not only the ability to overcome the disadvantages of standard sample preparation but also to increase the sensitivity of the analysis. All this makes magnetite nanoparticles a promising tool for the development of highly sensitive rapid analytical methods.

This paper presents the first development of LFIA for the determination of paraquat with magnetite nanoparticles. To increase the visual output of the assay results, magnetite nanoparticles are used in combination with gold nanoparticles. The analysis is based on the implementation of two types of conjugates—MNP-specific antibodies and GNP-anti-species antibodies. The first one is used in the competitive stage of the assay to form immune complexes, while the second helps overcome insufficient color intensity in the analytical zone and make the assay results more reliable.

## 2. Materials and Methods

### 2.1. Reagents and Materials

The chemicals that were used in the study include goat anti-mouse (anti-species) antibodies (Imtek, Moscow, Russia), iron (II) chloride (FeCl_2_), iron (III) chloride (FeCl_3_), chloroauric acid (HAuCl_4_ × 3H_2_O), sodium citrate, sodium azide, trimethyl ammonium chloride (TMACl), Tween-20, Triton 100, paraquat, propazin, fipronil, bifenthrin, cyproconazole, lambda-cyhalothrin (Sigma-Aldrich, St. Louis, MA, USA), and bovine serum albumin (BSA) (Boval Biosolutions, Cleburne, TX, USA). The specific immunoreagents for paraquat were obtained from Jiangnan University as described in [37]. Paraquat-BSA conjugates were synthesized using the mixed acid anhydride method, and anti-paraquat monoclonal antibodies were obtained using hybridoma technology. All other reagents were of analytical or chemical grade.

The working nitrocellulose membrane HF090 was from Millipore (Burlington, MA, USA) and the AP-045 adsorption membrane was from Advanced Microdevices (Ambala Cantt, Haryana, India).

### 2.2. Synthesis of MNPs

MNPs were synthesized as described in [38]. A total of 3.42 g FeCl_2_, 9.19 g FeCl_3,_ and 100 mL of 2 M HCl was added to 20 mL of water. Then, 300 mL of 2 M NaOH was added under vigorous stirring (860 rpm) using an Ekros-8300 overhead stirrer (Ekros, St. Petersburg, Russia). The reaction mixture was stirred for 30 min. The obtained nanoparticles were precipitated using a neodymium magnet (diameter 6 cm, height 3 cm, adhesion force 70 kg) and transferred to bidistilled water. To stabilize the surface, the magnetite nanoparticles were mixed with 50 mL 0.1 M TMACl. The synthesized preparation was stored at 4 °C.

### 2.3. Synthesis of GNPs

GNPs were synthesized as described in [39].

### 2.4. Characterization of MNPs and GNPs

Images of nanoparticles were characterized as described in [40].

To determine the hydrodynamic diameters (using dynamic light scattering (DLS)) and ξ-potential of the nanoparticles, a Zetasizer Nano ZSP (Malvern Instruments, Malvern, UK) nanoparticle analyzer was used. Measurements were conducted at 25 °C, with light scattering recorded at an angle of 173°. The results were processed using the Zetasizer software, version 7.11.

### 2.5. Conjugation of MNPs and GNPs with Antibodies

GNP conjugates with GAMI were synthesized as described in [40].

To obtain conjugate of MNPs with antibodies, the anti-paraquat antibodies were added to MNPs at a final concentration of 100 μg/mL. After 1 h of incubation with stirring, then BSA solution (10%) was added and incubated for 15 more minutes with constant stirring. The resulting conjugate of MNPs with specific antibodies was precipitated using a magnet and washed with 50 mM potassium phosphate buffer, pH 7.4, with 0.1 M NaCl (PBS).

### 2.6. ELISA for Paraquat

At the initial stage, the reactivity of the anti-paraquat monoclonal antibodies was characterized by the ELISA technique, considering their interaction with the antigen immobilized on the surface of the microplate wells. To assess the antibody affinity, paraquat detection was performed in a competitive ELISA scheme.

The solution of paraquat-BSA conjugate in 50 mM PBS (1 μg/mL, 100 μL) was added to the microplate wells and incubated for 1.5 h at 37 °C. The microplate wells were then washed four times using PBS with the addition of 0.05% Triton X-100 (PBST) (Sigma-Aldrich, St. Louis, MA, USA) to separate unbounded molecules and non-covalently immobilized paraquat-BSA conjugate.

**Figure 1:**
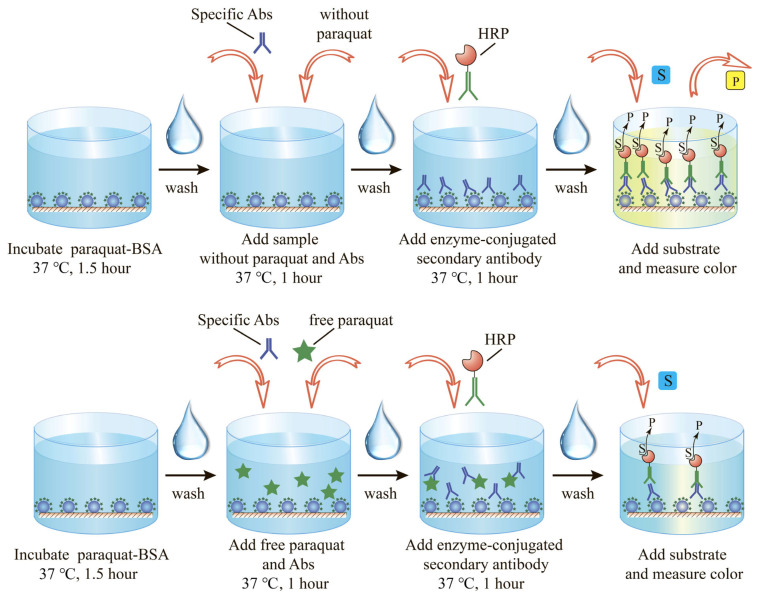
Scheme of ELISA for paraquat.

Next, 50 μL of paraquat (100 ng/mL to 0.02 ng/mL) was added. After that, 50 μL of monoclonal anti-paraquat antibodies (11 ng/mL) was added to each well and incubated for 60 min at 37 °C. The microplate was washed 3 times in PBS with Triton X-100. After this, 100 μL of goat anti-mouse antibodies (diluted 1:3000 in PBST) labeled with peroxidase was added and incubated for 1 h at 37 °C. After washing the microplate four times, 50 μL of 0.4 mM 3,3′,5,5′-tetramethylbenzidine (TMB) substrate solution with 0.01% H_2_O_2_ was added to the wells, incubated for 10 min, and the reaction was stopped by adding 50 μL of 1 M H_2_SO_4_. The scheme of the described analysis is given in Figure 1. The optical density of the reaction product was measured at 450 nm using a Zenyth 3100 microplate reader. The results of the performed ELISA are presented in the Supplementary Materials (Figure S1).

### 2.7. Construction of the Test Strips

The description of the test strip production is presented in the Supplementary Materials (Section S1.1).

### 2.8. Performing LFIA

To provide LFIA, paraquat (100–0.14 ng/mL) was added to conjugate of MP-anti-paraquat antibodies (31.25 μg/mL) in 30 µL PBS with 0.5% Tween-20 (PBST) or to orange, apples, and zucchini samples (dilution coefficient 2) in microplate wells. The test strips with immobilized paraquat-BSA conjugate were introduced into the wells in a vertical position for 5 min. After that, the test strips were replaced in the wells with GNP-anti-species antibodies conjugate (OD_520_ = 1.0) for 10 more min. Next, test strips were introduced to the wells with PBST to wash the excessive amount of conjugate (5 min). After the analysis had been completed, test strips were removed, placed on a horizontal surface, and then immediately scanned. All experiments were performed at room temperature.

### 2.9. Sample Preparation

The oranges, apples, and zucchini for the analyses were purchased from a local grocery store. All products were preliminarily separated from the peel and then grounded using a kitchen homogenizer. The resulting samples were filtered and used for the analyses.

### 2.10. Processing Test Strip Images and Calculating Assay Parameters

The description of the test strip processing and calculation of the results are presented in the Supplementary Materials (Section S1.2).

## 3. Results and Discussion

### 3.1. Assay Principle

The use of magnetite nanoparticles in LFIA provides several advantages that contribute to the increased sensitivity of the analysis. The enlarged particle surface area increases the concentration of binding molecules in the reaction mixture. This facilitates more efficient detection of low concentrations of the target analyte. Moreover, the use of an external magnetic field facilitates the efficient elimination of matrix impurities from the analyzed samples, thereby excluding non-specific influence on the assay outcomes. Thus, the use of magnetite nanoparticles in LFIA not only decreases the detection limit but also improves the efficiency of sample preparation and the accuracy of the analysis.

**Figure 2:**
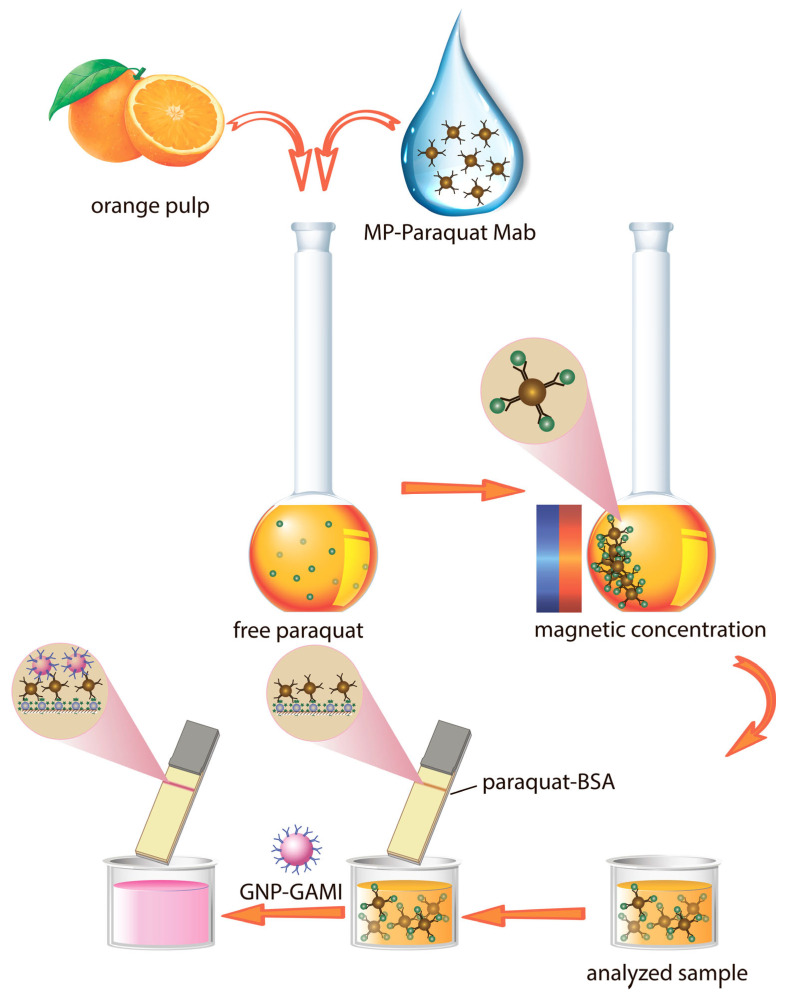
Scheme of the assay.

The proposed scheme of LFIA for paraquat includes the use of magnetite nanoparticles in combination with GNPs for the additional increase in color intensity in the analytical zone. The first stage of the LFIA involves the competitive determination of paraquat. MNPs conjugated with specific antibodies and the target analyte are added to the sample at various concentrations, while the paraquat-BSA conjugate is immobilized on the working membrane. When the test strip is immersed in the sample, competitive interaction occurs between the antigen and the hapten–protein conjugate for free antibody-binding sites. This leads to suppression of the colored complex formation in the analytical zone in case the antigen concentration in the sample exceeds the acceptable limits. Since the use of MNPs conjugated with specific antibodies complicates the reliable visual interpretation of the results of the analysis, at the second stage, a conjugate of GNPs with anti-species antibodies is added to provide additional contrast to the analytical zone (Figure 2).

### 3.2. Characterization of Nanoparticles

**Figure 3:**
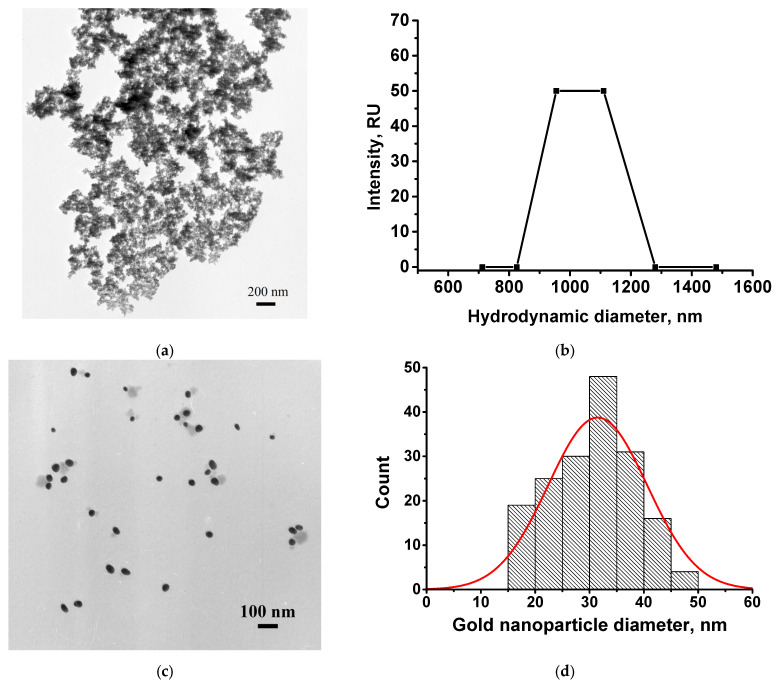
Characterization of MNPs (**a**,**b**) and GNPs (**c**,**d**): TEM micrographs (**a**,**c**) and histograms of diameter distributions (**b**,**d**). The red curve is an approximation by a Gaussian distribution.

TEM micrographs of the synthesized MNPs indicate the formation of aggregates with an average diameter of 100 ± 12 nm. The values of the hydrodynamic diameter determined by DLS were 920 ± 68 nm (Figure 3b). MNPs in solution primarily form aggregates, which is shown in Figure 3a, obtained by TEM. However, TEM also shows the individual particles being approximately 10 nm in size. The measurements of DLS confirm the aggregation state of MNPs in the solution. DLS reflects integral properties of the aggregates and so its data significantly exceed the measurements for individual nanoparticles that can be recognized in TEM images. The aggregation stability of magnetite nanoparticles was assessed using the ξ-potential, which was found to be −7.9 ± 0.5 mV. The given z-potential parameter reflected properties of pure particles. The surface charge of the obtained MNPs was significantly lower than that of the gold nanoparticles, making them less stable and prone to aggregation. However, the aggregation of MNPs enhances the efficiency of their use for magnetic separation. Therefore, a balance between stability and the magnetic properties of the particles should be reached. The obtained MNPs were conjugated with antibodies and further stabilized with BSA. These procedures simultaneously functionalize and stabilize the particles, preventing their further aggregation. Thus, the functionalization of nanoparticles with antibodies was found to be sufficient to obtain stable conjugates.

The average diameter of the synthesized GNPs was 31.5 ± 9.3 nm (*n* = 171, the minimum value was 16.8 nm, and the maximum value was 65.2 nm) with a degree of ellipticity of 1.25 ± 0.07 (Figure 3c,d).

### 3.3. Estimation of the Conditions for LFIA

To estimate the optimal conditions for LFIA performance, we determined the dependence of color intensity in the analytical zone and detection limit on the concentrations of the test system components.

#### 3.3.1. Choice of Concentration for MNP-Specific Antibodies Conjugate

**Figure 4:**
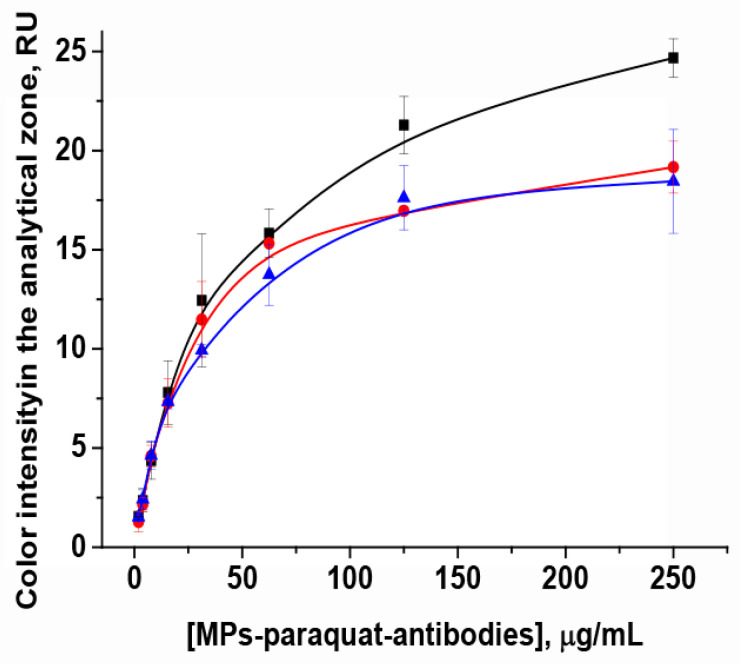
Dependences of color intensity in the analytical zone on concentration of MNP-specific antibodies conjugate and on concentration of paraquat-BSA conjugate immobilized in the analytical zone. Black, red, and blue curves correspond to paraquat-BSA conjugate concentrations of 2.0 mg/mL, 1.0 mg/mL, and 0.5 mg/mL, respectively (*n* = 3).

First, the concentrations of the magnetic particle conjugate with specific antibodies and the paraquat-BSA conjugate were varied (Figure 4). As can be seen, color intensity in the analytical zone reduces with the reduction in particle concentration. For concentrations of paraquat-BSA conjugate equal to 2.0 mg/mL, the highest intensity was observed using maximum amounts of MNP conjugate (31.25–250 µg/mL). When using 1.0 mg/mL and 0.5 mg/mL of paraquat-BSA conjugate concentrations, the staining in the analytical zone was moderate. For further work, we chose the concentration of MNP conjugate of 31.25 µg/mL, while the response of color intensity corresponds to average intensity and to the moderate reagent consumption, which will also likely provide a lower detection limit of the assay.

#### 3.3.2. Choice of Concentration for Paraquat-BSA Conjugate Concentration Applied to the Analytical Zone

**Figure 5:**
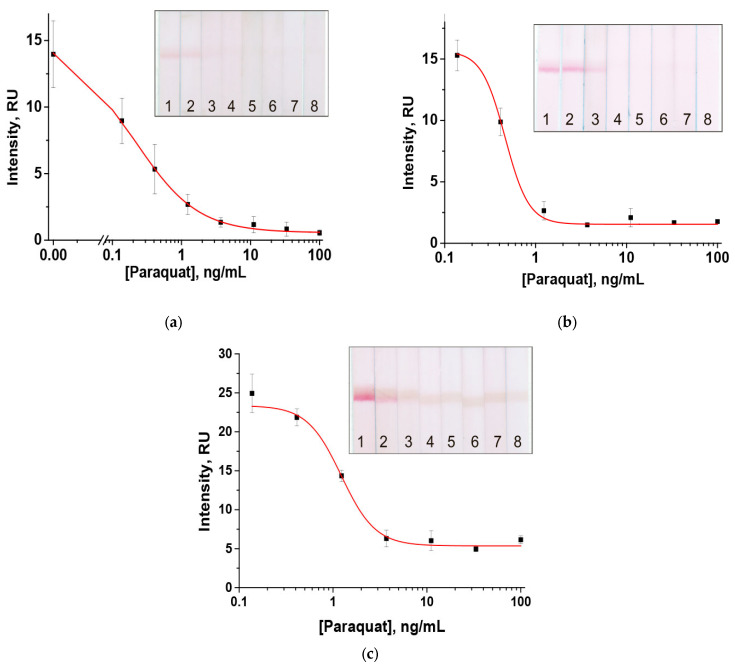
Concentration dependences of paraquat LFIA for different paraquat-BSA conjugate concentrations applied to the analytical zone: 0.5 mg/mL (**a**), 1.0 mg/mL (**b**), and 2.0 mg/mL (**c**). The concentrations of paraquat in the samples for the test strips numbered as 1–8 on the inserts were 0.0; 0.05; 0.14; 0.4; 1.2; 3.7; 11; 33; and 100 ng/mL, respectively (*n* = 3).

We then tested the dependence of the detection limit on the concentration of paraquat-BSA in the analytical zone. To reduce the excessive amount of conjugate on the test strips and make their appearance clearer, we performed the analysis with the additional washing step in PBST. However, this modification of the assay protocol did not lead to a reliable change in the analytical characteristics of the test system. When using a paraquat-BSA conjugate with a concentration of 2.0 mg/mL, the visual limit of detection exceeds the established maximum residue level for paraquat (100 ng/mL). Thus, the utilization of such a parameter is not applicable. Performing the assay with lower hapten–protein conjugate concentrations (0.5 mg/mL and 1.0 mg/mL) resulted in visual detection limits of 1.2 ng/mL for both cases, while the instrumental limits of detection were 0.02 ng/mL and 0.2 ng/mL for paraquat-BSA conjugate concentrations of 0.5 mg/mL and 1.0 mg/mL, respectively (Figure 5). For further work, a paraquat-BSA conjugate concentration of 1.0 mg/mL was chosen.

### 3.4. Testing the Developed LFIA in Orange Samples

**Figure 6:**
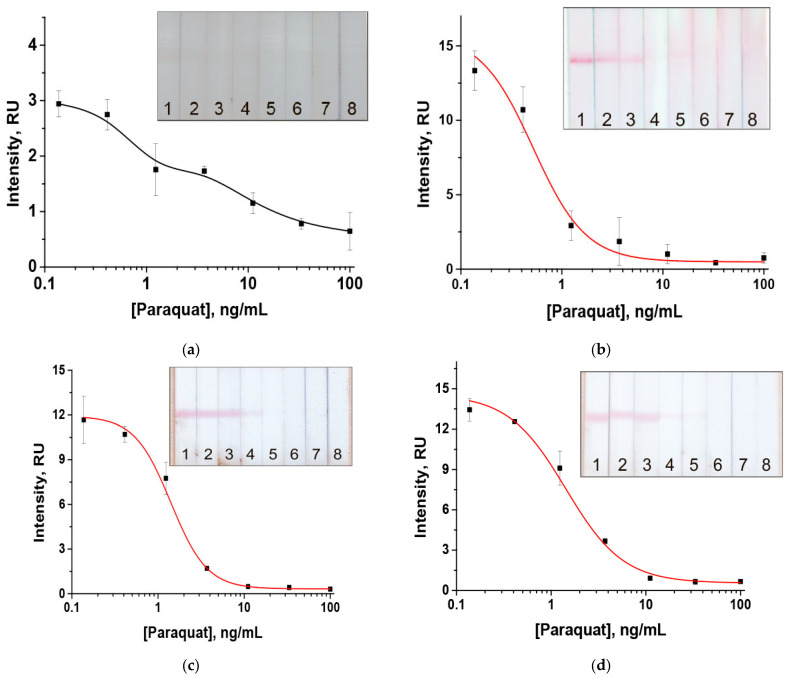
LFIA for paraquat with MP (**a**) and with GNP enhancement in oranges (**b**); apples (**c**), and zucchini (**d**). The concentrations of paraquat in the samples for the test strips numbered as 1–8 on the insert were 0.0; 0.05; 0.14; 0.4; 1.2; 3.7; 11; 33; and 100 ng/mL, respectively (*n* = 3).

The developed LFIA was tested for samples of oranges with paraquat concentrations from 0.14 to 100 ng/mL (Figure 6). The obtained detection limits were as follows: 1.2 ng/mL (visual) and instrumental—0.12 ng/mL (0.24 µg/kg) for oranges, 1.2 ng/mL (visual) and instrumental—0.31 (0.62 µg/kg) for apples, and 3.7 ng/mL (visual) and instrumental—0.48 ng/mL (0.96 µg/kg) for zucchini, which is acceptable in comparison with the established maximal permissible levels for paraquat (0.05–0.1 mg/kg) [15]. The use of magnetite nanoparticles combined with gold nanoparticles in the assay increases its color intensity for visual detection by five times (2.9 RU compared to 15.3 RU), which makes the analysis results more reliable. Recoveries for paraquat were estimated from 96 to 120% (Table 1).

### 3.5. Selectivity Testing of the Developed LFIA

**Figure 7:**
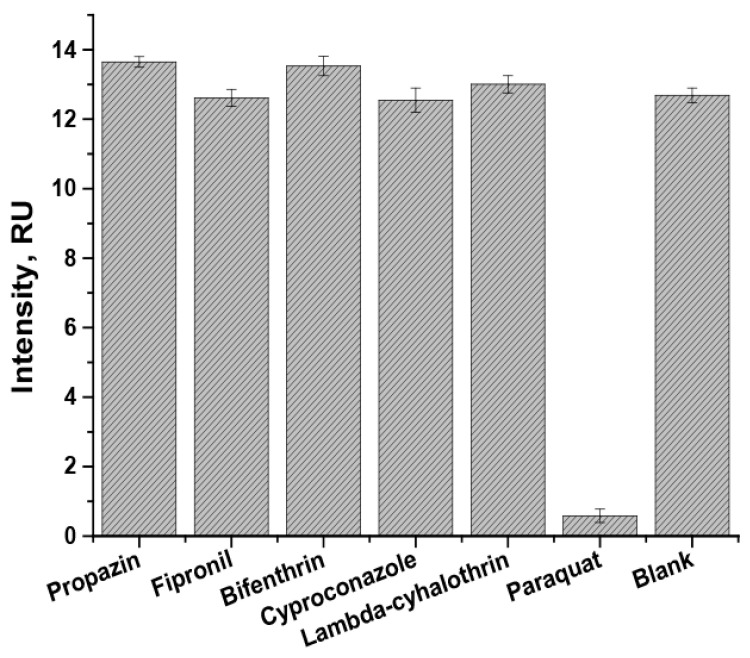
Selectivity of the developed LFIA for paraquat. Dependencies of color intensity of the analytical zone on non-target analytes binding with specific antibodies.

The selectivity of the assay was tested with pesticides from other groups (propazine, fipronil, bifenthrin, cyproconazole, and lambda-cyhalothrin). First, when paraquat was added to the sample (100 ng/mL) with the conjugate of MNPs and specific antibodies, it filled the binding sites of the antibodies. The reaction between paraquat and specific antibodies prevents the possibility of the label’s binding with the paraquat-BSA conjugate in the analytical zone of the test strip. Therefore, there was no visible coloration of the analytical zone (Figure 7).

In the case when one of other pesticides mentioned above (250 ng/mL) was added to the sample with the conjugate of MNPs and specific antibodies, the color intensity in the analytical zone did not differ from the case with the addition of pure buffer (blank preparation without any cross-reactants). This means that the tested pesticides do not bind with the antibodies against paraquat.

### 3.6. Performing LFIA for Paraquat with Magnetic Concentration

To reduce the detection limit of the assay, magnetic preconcentration of the antigen using a magnetic particle-specific antibody conjugate was used. To do so, low concentrations of antigen (0.04 ng/mL; 0.3 ng/mL; 0.5 ng/mL; and 0.7 ng/mL) and magnetic particle conjugate were added to samples of varying volumes, incubated for 5 min, and then precipitated using an external magnetic field. The supernatant was then removed, the pellet was resuspended in a working buffer (PBST), and paraquat was determined in the test sample. The experiments were conducted at room temperature for sample volumes of 1, 5, and 10 mL with resuspension in 30 µL, which corresponds to 33-, 167-, and 333-fold concentrations (Table 2).

From the obtained data, it is evident (Table 2) that when concentrating, for example, 33 times, the visual detection limit decreases to 0.04 ng/mL. Concentrations below the visual detection limit when concentrating by 33–333 times become possible to qualitatively evaluate.

### 3.7. Comparison of the Developed and Earlier Described LFIAs for Paraquat

Among the recently developed LFIAs for paraquat, most systems are based on the use of gold nanoparticles. It is shown that the application of the developed approach of combining MNP and GNP conjugate allows for the achievement of a lower detection limit in comparison with other works (Table 3). The combination of MNPs as a tool of concentration and GNPs as a tool of intensive optical labeling provides the possibility to form functionalized complexes and to decrease the detection limit of the assay. The implementation of this newly proposed approach in lateral flow immunoassay on the example of paraquat detection demonstrated its efficiency and potential as a powerful tool for detecting various hazardous compounds, ensuring effective food safety control.

## 4. Conclusions

The LFIA for paraquat detection has been developed based on magnetite nanoparticles and additional gold nanoparticle contrasting. Among other GNP-based LFIAs for paraquat, this is the first development utilizing magnetite nanoparticles. The reached detection limit was in the range of 1.2–3.7ng/mL (visual) and 0.12–0.48 ng/mL (instrumental). It was shown that magnetite nanoparticles provide efficient and simple work with large sample volumes, decreasing the detection limit. The implementation of the assay, along with the magnetic concentration stage, allows for the collection of paraquat from large sample volumes and enables detection at concentrations up to two orders of magnitude lower. In perspective, magnetic-based LFIA can be used in combination with not only colorimetric but also fluorescent and dye nanoparticles to provide more efficient analytical characteristics.

## Figures and Tables

**Table 1:** Recoveries of paraquat in oranges, apples, and zucchini for LFIA with magnetite nanoparticles (*n* = 3).

Added, ng/mL	Detected, ± SD, ng/mL	Recovery, %
	Orange	
0.25	0.26 ± 0.07	104
0.5	0.48 ± 0.09	96
1.0	1.1 ± 0.3	110
	Apple	
0.75	0.73 ± 0.08	97
1.0	1.2 ± 0.4	120
2.0	2.1 ± 0.4	105
	Zucchini	
0.75	0.77 ± 0.12	103
1.0	1.1 ± 0.3	110
2.0	2.3 ± 0.5	115

**Table 2:** Detection of paraquat with magnetic concentration.

Paraquat Added, ng/mL	Paraquat Detected, ng/mL						
	Without Concentration	33-Fold Concentration		167-Fold Concentration		333-Fold Concentration	
		Theoretical	Founded	Theoretical	Founded	Theoretical	Founded
0.7	0.73 ± 0.05	23.1	21.1 ± 1.2	116.9	113.2 ± 5	233.1	231.1 ± 6
0.5	0.52 ± 0.04	16.5	16.3 ± 1.3	83.5	78.4 ± 4	166.5	160.5 ± 9
0.3	0.31 ± 0.2	9.9	8.7 ± 0.4	50.1	45 ± 3	99.9	93 ± 7
0.04	- *	1.32	1.3 ± 0.1	6.68	6.9 ± 2	13.32	12.9 ± 4

*—the concentration is outside of the working range.

**Table 3:** Comparative characteristics of LFIAs for paraquat detection.

Used Nanoparticles	Binding Reactant	Matrix	Limit of Detection	Reference
GNPs	Antibody	Water	0.25–1 ng/mL	[37]
GNPs	Antibody	Herbicide products	20 ng/mL (visual)	[41]
GNPs	Antibody	Soybean, potato, and banana	4 ng/mL (visual); 1.69 ng/mL (instrumental)	[42]
GNPs	Antibody	Serum and urine	20 ng/mL (visual)	[43]
GNPs	Antibody	Water, whole blood, plasma, and urine	10 ng/mL (visual)	[44]
Fluorescent microspheres	Nanobody	Chinese cabbage, pear, blood, urine, rice, and corn	3 ng/mL (visual); 0.009 ng/mL (instrumental)	[45]
GNPs	Aptamer	Cucumber, apple, and Chinese cabbage	30 ng/mL (visual); 4.28 ng/mL (instrumental)	[46]
MNPs-based LFIA with a combination of GNPs	Antibody	Oranges	1.2 ng/mL (visual); 0.12 ng/mL (instrumental)	This study

## Data Availability

The data presented in this study are available on request from the corresponding author.

## Supplementary Materials

The following supporting information can be downloaded at https://www.mdpi.com/article/10.3390/toxics14010002/s1. Section S1. Materials and Methods; Section S2. Characterization of immunoreagents; Figure S1: Estimation of antibodies’ reactivity (a) and detection limit of paraquat (b) in ELISA.
