# Supplementary material for: Enhanced Lateral Flow Immunoassay for Pesticide Paraquat Based on Combining Magnetite and Gold Nanoparticles
Source: Toxics. 2025 Dec 19;14(1):2. doi: 10.3390/toxics14010002 (PMC12845944; doi:10.3390/toxics14010002)
Supplement: Supplementary file 1 [file toxics-14-00002-s001.zip › toxics-3987234-supplementary.pdf]

Supplementary Information

Enhanced lateral flow immunoassay for pesticide paraquat based on combining magnetite and gold nanoparticles

Lyubov V. Barshevskaya, Nadezhda A. Taranova, Dmitry V. Sotnikov, Chuanlai Xu[2], Anatoly V. Zherdev, Boris B. Dzantiev*

A.N. Bach Institute of Biochemistry, Research Center of Biotechnology of the Russian Academy of Sciences, Leninsky prospect 33, 119071 Moscow, Russia

[2] International Joint Research Laboratory for Biointerface and Biodetection, State Key Lab of Food Science & Technology, and School of Food Science and Technology, Jiangnan University, Wuxi, China

* Correspondence: dzantiev@inbi.ras.ru, Tel.: +7-495-954-31-42

Section S1. Materials and Methods

S1.1 Construction of the test strips

Millipore HF090 working nitrocellulose membrane was used to form analytical zone by applying paraquat-BSA solution in PBS (0.5; 1.0; 2.0 mg/mL; application consumption 1 μL/mm) using an IsoFlow automatic dispenser (Imagene Technology, Lebanon, NH, USA). The working membrane was then dried at 20–22 °C for 24 h. A multimembrane composite (including working membrane and AP-045 adsorption membrane) was assembled, cut into 3.5 mm-wide strips using an Index Cutter-1 automatic guillotine cutter (A-Point Technologies Brea, CA, USA) and stored at room temperature in a sealed package containing silica gel.

S.1.2 Processing test strip images and calculating assay parameters

After LFIA, the test strips were scanned with a Canon Lide 90 flatbed scanner (Canon, Tokyo, Japan) at 600 dpi resolution without contrast and color correction mode and analyzed with the Total Lab software (Nonlinear Dynamics, Newcastle upon Tyne , UK). Line coloring intensities were presented as the same relative units for all data within the article.

The dependences of coloration intensity in the analytical zone on the antigen concentration in the sample (x) were approximated by the Origin 9.0 software (OriginLab, Northampton, MA, USA) using a 4-parameter sigmoid function:

$$y = (a - b)/[1 + (x/c)^d] + b,$$

where a = maximal signal, b = minimal signal, c (or IC50) = the antigen concentration at which the decrease of y is 50% of its range of changes, and d = the slope of the approximating curve at point c.

The antigen concentration, corresponding to the disappearance of color in the analytical zone, was taken as the visual detection limit.

Section S2. Characterization of immunoreagents

Using ELISA, we assessed the reactivity of antibodies based on their interaction with the antigen immobilized on the microplate surface. The concentration of antibodies corresponding to OD_{450} = 1.0 was selected to determine the target analyte. According to Fig.S1a, the selected concentration of monoclonal antibody to paraquat was 11 ng/mL.

To estimate the detection limit of paraquat, a competitive interaction was performed in ELISA (Fig. S1b). The detection limit of paraquat was 0.03 ng/ml with working range of 0.03-0.27 ng/mL.

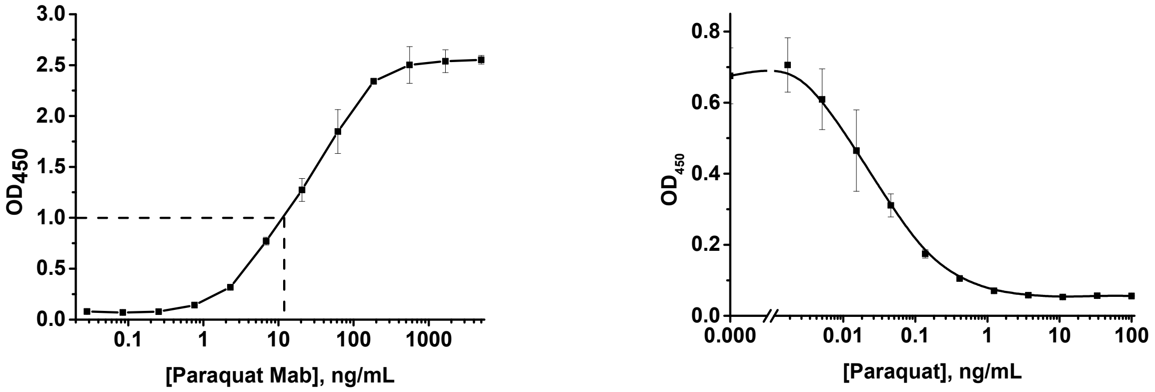

(**a**) (**b**)

Figure S1. Estimation of antibodies reactivity (a) and detection limit of paraquat (b) in ELISA.
